# Spatial Characterization of Radio Propagation Channel in Urban Vehicle-to-Infrastructure Environments to Support WSNs Deployment

**DOI:** 10.3390/s17061313

**Published:** 2017-06-07

**Authors:** Fausto Granda, Leyre Azpilicueta, Cesar Vargas-Rosales, Peio Lopez-Iturri, Erik Aguirre, Jose Javier Astrain, Jesus Villandangos, Francisco Falcone

**Affiliations:** 1 Electrical and Electronic Engineering Dept., Universidad de las Fuerzas Armadas ESPE, Sangolquí 171-5-231B, Ecuador; flgranda@espe.edu.ec; 2 School of Engineering and Sciences, Tecnologico de Monterrey, Monterrey 64849, Mexico; cvargas@itesm.mx; 3 Electrical and Electronic Engineering Dept, Public University of Navarre, Pamplona 31006, Spain; peio.lopez@unavarra.es (P.L.-I.); erik.aguirre@unavarra.es (E.A.); francisco.falcone@unavarra.es (F.F.); 4 Mathematical Engineering and Computer Science Department, Public University of Navarre, Pamplona 31006, Spain; josej.astrain@unavarra.es (J.J.A.); jesusv@unavarra.es (J.V.); 5 Institute of Smart Cities, Public University of Navarre, Pamplona 31006, Spain

**Keywords:** wireless sensor networks (WSN), 3D ray launching, Vehicular ad-hoc Networks (VANET), vehicle-to-Infrastructure communication (V2I), path loss, coherence bandwidth

## Abstract

Vehicular ad hoc Networks (VANETs) enable vehicles to communicate with each other as well as with roadside units (RSUs). Although there is a significant research effort in radio channel modeling focused on vehicle-to-vehicle (V2V), not much work has been done for vehicle-to-infrastructure (V2I) using 3D ray-tracing tools. This work evaluates some important parameters of a V2I wireless channel link such as large-scale path loss and multipath metrics in a typical urban scenario using a deterministic simulation model based on an in-house 3D Ray-Launching (3D-RL) algorithm at 5.9 GHz. Results show the high impact that the spatial distance; link frequency; placement of RSUs; and factors such as roundabout, geometry and relative position of the obstacles have in V2I propagation channel. A detailed spatial path loss characterization of the V2I channel along the streets and avenues is presented. The 3D-RL results show high accuracy when compared with measurements, and represent more reliably the propagation phenomena when compared with analytical path loss models. Performance metrics for a real test scenario implemented with a VANET wireless sensor network implemented ad-hoc are also described. These results constitute a starting point in the design phase of Wireless Sensor Networks (WSNs) radio-planning in the urban V2I deployment in terms of coverage.

## 1. Introduction

According to the World Health Organization, about 1.25 million people die each year as a result of road traffic crashes and, without action, these have been predicted to rise to become the 7th leading cause of death by 2030 [1]. In 2014, motor-vehicle traffic-related injuries accounted for 16.9% of all injury deaths in United States [2]. In Mexico, during 2015, the traffic accidents in urban and suburban zones caused approximately 111,000 victims between drivers, passengers, pedestrians, cyclists and others [3]. The safety of vehicle occupants and nonvehicle people has been increasing the commercial, governmental and research interest for technologies that enhance the ability of vehicles to sense, process, act on and communicate data to other travelers, vehicles or infrastructure. For instance, the private sector is developing full autonomous vehicles [4,5], the U.S. Department of Transportation announced new Federal Highway Administration (FHWA) Vehicle-to-Infrastructure guidance that will accelerate the deployment of V2I communication systems [6]. Research projects at academic organizations are being conducted on Intelligent Transportation System (ITS) [7,8]. These ongoing efforts are encompassed in the area of ITS where the connected vehicles research includes three major approaches to communication: Vehicle-to-Vehicle (V2V), Vehicle-to-Infrastructure (V2I) and Vehicle-to-Pedestrian (V2P). The V2I Communications is the bi-directional wireless exchange of data (control and information) between vehicles and Road Side Units (RSUs). In 1999, the U.S. Federal Communication Commission (FCC) allocated a 75 MHz spectrum at 5.9 GHz to be used as V2V and V2I communications known as Dedicated Short Range Communications (DSRC), and the IEEE 802.11p standard [9] was developed for operation at 5.9 GHz. In Europe, governments have licensed the 5.9 GHz spectrum, while Japan has licensed the 5.8 GHz spectrum for vehicular communications.

V2I has been considered for supporting all range of applications, not only safety critical applications [10], such as stop sign violation warning (SSVW), railroad crossing violation warning (RCVW), and oversize vehicle warning (OVW), but also on-car multimedia streaming, [11], and gaming. Other applications that can take advantage of V2I communications are: traffic information systems (TIS), intersection collision warning systems (ICWS) [12], intelligent traffic management systems (ITMS), vehicle collision prediction [13], parking management systems (PMS), [14], cooperative advanced cruise control (CACC) [15], short-term traffic prediction, platooning, lane detection, obstacle warning, target tracking, etc. Smart cities will be able to take advantage of the aforementioned application and integrate V2I information from ITS into vehicle star/stop features navigation systems, predictive services, etc., in order to improve efficiency, drive time, traffic management or, driver, passenger and pedestrian wellness. It is worth noting that the deployment of the V2I application and others aimed at the convergence of sensing, infrastructure and information technologies into the ITS rely on the accurate channel characterization and the understanding of the propagation phenomena.

In the V2I urban environment, a combination of different object types such as buildings, vehicles (both static and mobile), and vegetation, as well as their number, size, and density, has a profound impact on the radio propagation. Some propagation impairments are analogous at the presented in short-range outdoor radio communication systems [16]: Reflection from, diffraction around and transmission loss through objects (influence of vegetation, building entry loss, cars, trees, pedestrians, etc.), external environment, which gives rise to issues such as temporal and spatial variation of path loss and multipath effects from reflected and diffracted components of the wave. A measurement campaign is the ideal method to quantify the effect of this phenomenon, however, the technological, economical and human resources are not always available. Alternatives such as analytical models, stochastic models and ray tracing approaches are used to capture these propagation impairments. For example, Cardote et al. propose [17] a statistical channel model for VANET simulation based on the observation of data from real-world experiments, obtained from an IEEE 802.11p/WAVE compliant testbed. Fazio et al. present [18] a new approach for modeling the wireless channel of VANETs communications based on Discrete Time Markov Chain (DTMC) modeling. Akhtar et al. provide [19] a realistic analysis of the VANET topology characteristics over time and space for a highway scenario, using various key metrics of interest, including node degree, neighbor distribution, number of clusters and link duration. Nilsson et al. present [20] a measurement based analysis of multilink shadowing effects in a V2V communication system with cars as blocking objects.

Nonetheless, some analytical models that assume exponential path loss are not appropriate if scenarios with buildings are investigated [12]. Stochastic models that describe the channel characteristics from a macroscopic point of view might lead to severe deviations from realistic behavior and application as safety cannot be modeled accurately. The ray-tracing model has been recognized as an accurate tool to model propagation phenomena [21,22,23]. There are some scientific works for V2I signal propagation analysis for urban areas using deterministic tools, such as ray launching (RL). Ray-tracing models yield an excellent approximation of the real-world propagation with a reasonable computational complexity, and even with limited details and materials characterization can still result in accurate levels of prediction of both geometry and shadow levels [24], and can serve as a tool to simulate radio shadowing environments. An empirical study of propagation in urban areas for V2I is presented by Shemshaky et al. [25]. Wang et al. [26] make use of an in-house 3D ray-tracing software to present a statistical peer-to-peer channel model for outdoor urban environments. The use of deterministic in-house or commercial 3D ray-tracing software to predict power as well as time, frequency and spatial dispersion in the radio channel in urban or V2X scenario is reported by Rodriguez et al. [24] and Campolo et al. [27].

In this work, a deterministic 3D Ray Launching (3D-RL) algorithm, based on Geometrical Optics (GO) and Geometrical Theory of Diffraction (GTD) whose operating mode of the algorithm was previously published by Azpilicueta et al. [28] and validated in transportation systems [29], was used to analyze some of the V2I propagation phenomenon in an urban environment at 5.9 GHz. The scenario, a typical urban environment (park, avenues, streets, buildings, roundabout, etc.) was characterized with high level detail (objects, materials, geometry of obstacles, electromagnetic properties, etc.), in order to obtain accurate results. A detailed exploratory analysis that considers the transmitter-receiver distance, the Line-of-Sight (LoS) and Non-Line-of-sight (NLoS) conditions, large-scale path loss, multipath metrics, the comparison of measurements versus the 3D-RL simulation and analytical models, is presented in a holistic form and constitutes an important contribution. Volumetric power estimations enable to obtain coverage/capacity ratios which aid in network deployment, whereas time domain estimation, such as volumetric power delay profile and delay spread values provide insight in equalization requirements as well as on the use and parameterization of adaptive modulation and coding schemes. Coverage/Capacity relations are introduced, providing node density values which aid in network deployment phases. Main contributions of this work include deterministic hybrid simulation techniques as a useful tool in system level analysis, the combination of physical layer with application-based results and the implementation of a functional vehicular testbed to analyze a real context aware environment.

The paper is arranged as follows: 3D-Ray Launching (3D-RL) technique is described in Section 2. The characterization and scenario simulation parameters are explained in Section 3. Section 4 presents the results of the received power, large-scale path loss analysis and multipath metrics. A validation of the real measurement versus simulation results and analytical models is presented in Section 5. Section 6 describes a real VANET test deployment scenario, in which an ad-hoc software architecture as well as wireless motes with input/output capabilities have been analyzed. Conclusions and future work are given in Section 7.

## 2. Background

### Ray Launching (RL) Technique

An in-house developed 3D-RL code has been used to analyze the electromagnetic propagation behavior of the considered scenario. The algorithm is based on GO and GTD, with its uniform extension, the Uniform GTD (UTD). The main principle of the RL techniques is that the wave front of the radiated wave is identified with a number of rays that propagate in the space according to optic and electromagnetic theories. Each ray propagates in space as a single optic ray.

GO approaches considers only direct, reflected and refracted rays, leading to the existence of abrupt areas, corresponding to the boundaries of the regions where these rays exist. This problem was initially solved by Keller [30] by introducing diffraction phenomenon. Keller stated that the diffracted rays behave as GO rays once they leave the edge, and their path can be obtained by generalizing Fermat’s principle [31]. Nevertheless, the original formulation developed by Keller presented some problems in the shadow and reflection boundaries where the GO fields present discontinuities. These problems were solved with uniform solutions to the asymptotic expressions that Keller used to develop his theory. The first one was the UTD, which has been, historically, the most widely used, and, therefore, has been selected to be implemented in the in-house developed 3D-RL code.

The RL technique methodology is based on the fact that a transmitter launches thousands of test rays in a solid angle and, by searching for the rays arriving at the receiver, the true path is determined. These methods are precise but are time-consuming due to inherent computational complexity. Their combination with Uniform Theory of Diffraction (UTD) [32] is frequently applied to radio coverage prediction [33,34,35]. These techniques potentially represent the most accurate and versatile methods for urban and indoor multipath propagation characterization.

The incident electric field (E_i_) created by an antenna at a distance *r* in the free space can be calculated by [36],

(1) $$E_{i}^{⊥∥}=\sqrt{\frac{P_{\mathrm{rad}}D_{t}\left(\theta_{t},\phi_{t}\right)\eta_{0}}{2\pi }}\frac{e^{-{j\beta }_{0}r}}{r}X^{⊥∥}L^{⊥∥},$$

where $P_{\mathrm{rad}}$ is the radiated power with a directivity $D_{t}\left(\theta_{t},\phi_{t}\right)$, where the sub-index t refers to the transmitted angle, and polarization ratio $\left(X^{⊥},X^{∥}\right)$. $\beta_{0}=2{\pi f}_{c}\sqrt{\varepsilon_{0}\mu_{0}}$, $\varepsilon_{0}=8.854\times 10^{-12}$, $\mu_{0}=4\pi \times 10^{-7}$ and $\eta_{0}=120\pi .$ $L^{⊥∥}$ are the path loss coefficients for each polarization. The parameter j in Equation (1) refers to the complex number.

A reflected and a transmitted ray are created when the rays impinge with an obstacle in its path, with new angles provided by Snell’s law [37]. When a ray hits an edge, a new family of diffracted rays is created provided by formulation of the UTD. The main purpose of these diffracted rays is to remove field discontinuities and to introduce proper field corrections, especially in the zero-field regions predicted by GO. The diffracted field is calculated by [38],

(2) $$E_{\mathrm{UTD}}=e_{0}\frac{e^{-{\mathrm{jks}}_{1}}}{s_{1}}D^{⊥∥}\sqrt{\frac{s_{1}}{s_{2}\left(s_{1}+s_{2}\right)}}e^{-{\mathrm{jks}}_{2}}$$

where $s_{1}$ and $s_{2}$ are the distances from the source to the edge and from the edge to the receiver point, respectively. $D^{⊥∥}$ is the diffraction coefficients given by [38,39].

Spatial resolution is also considered a uniform three-dimensional hexahedral mesh. Parameters such as frequency of operation, radiation patterns of the antennas, number of multipath reflections, and spatial and angular resolution can be taken into account. Furthermore, all the material properties for all the elements within the scenario are considered, given the dielectric constant and the loss tangent at the frequency range of operation of the system under analysis.

The algorithm is divided into three phases:

1. Phase 1: Creating the scenario. This phase sets the scenario, considering all the obstacles within the environment, and the transmitters and receivers.
2. Phase 2: Simulation of RL in 3D. In this phase, rays are launched from each transmitter, keeping the parameters in each position in space.
3. Phase 3: Analysis of the results. In this phase, the values are obtained from the simulation to calculate the desired parameters.

The detailed operating mode of the algorithm has been previously published [28], and it has been used and validated in different environments [40,41,42], including urban and city environments [21].

## 3. Simulated Urban Scenario

Deterministic models, such as 3D-RL, have the advantage that computer simulations are easier to perform than extensive measurement campaigns, however, the accuracy of the 3D-RL results depends on the detailed geographical description of the scenario together with the electromagnetic properties of the interacting objects. This section describes the modeled scenario with its geometry, objects, points of interest and the main simulation parameters.

**Figure 1:**
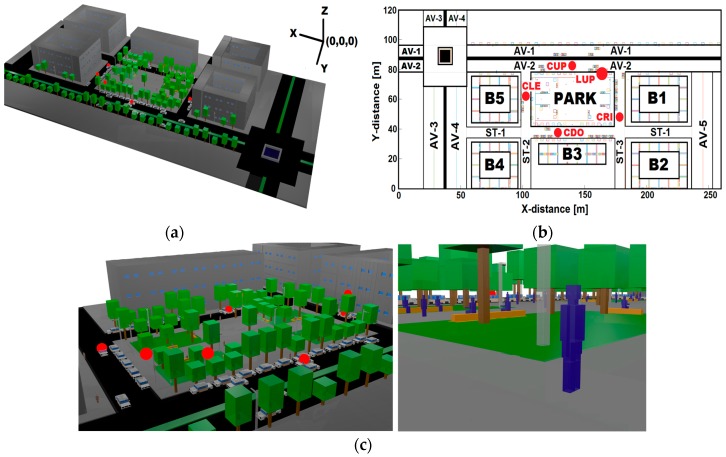
Scenario: (**a**) 3D frontal-view; (**b**) 2D back-view; and (**c**) close-up view.

Figure 1a depicts a 3D aerial-view of the modeled scenario where buildings, streets and trees are shown. Figure 1b displays a 2D aerial-view which identifies the main points and areas of interest and its relative position (x, y), according to Table 1. Figure 1c shows a close-up view of some scenario elements used in the 3D-RL simulation.

The 3D-RL incorporates a database of geographic data such as vegetation, terrain, buildings, park-benches, cars, pedestrians, avenues, streets, roundabouts and sidewalks of an urban scenario modeled and characterized as a replica of Plaza de Gongora (42°47′52.72′′ N, 1°38′21.14′′ W) located in Pamplona, Spain. The area encompasses 624,000 m^3^ (260 m × 120 m × 20 m) and includes: one park, one roundabout (with fountain), three secondary streets, five motorway avenues, five buildings (20 m high), eight park benches, eight medium lampposts (7 m high), 13 high lampposts (10 m high), 16 pedestrians, 45 cars, 100 trees and many sidewalks; each of them has its own electromagnetic characteristics for the analysis in the 3D-RL algorithm.

The simulation parameters are registered in Table 2. The simulation information was gathered from 624,000 cuboids of 1 m × 1 m × 1 m evenly distributed. The transmitter antenna (Tx) was located at 3.5 m height in the upper lamppost (LUP). The receiver (Rx) antenna was placed at 1.5 m height. The transmitted power was settled at −10 dBm and the Received Signal Threshold (RST) at −120 dBm. The reference distance d_o_ = 1 m.

## 4. Results

This section comprises the 3D-RL analysis of the Received Signal Strength (RSS); the large-scale spatial path loss where parameters such as Path Loss Exponent (PLE) and Standard Deviation (STD) obtained from the 3D-RL simulation; and the multipath metrics such as Power delay Profile, Mean excess delay, Root-Mean-Square Delay Spread and Coherence Bandwidth.

### 4.1. Received Signal Strength (RSS)

**Figure 2:**
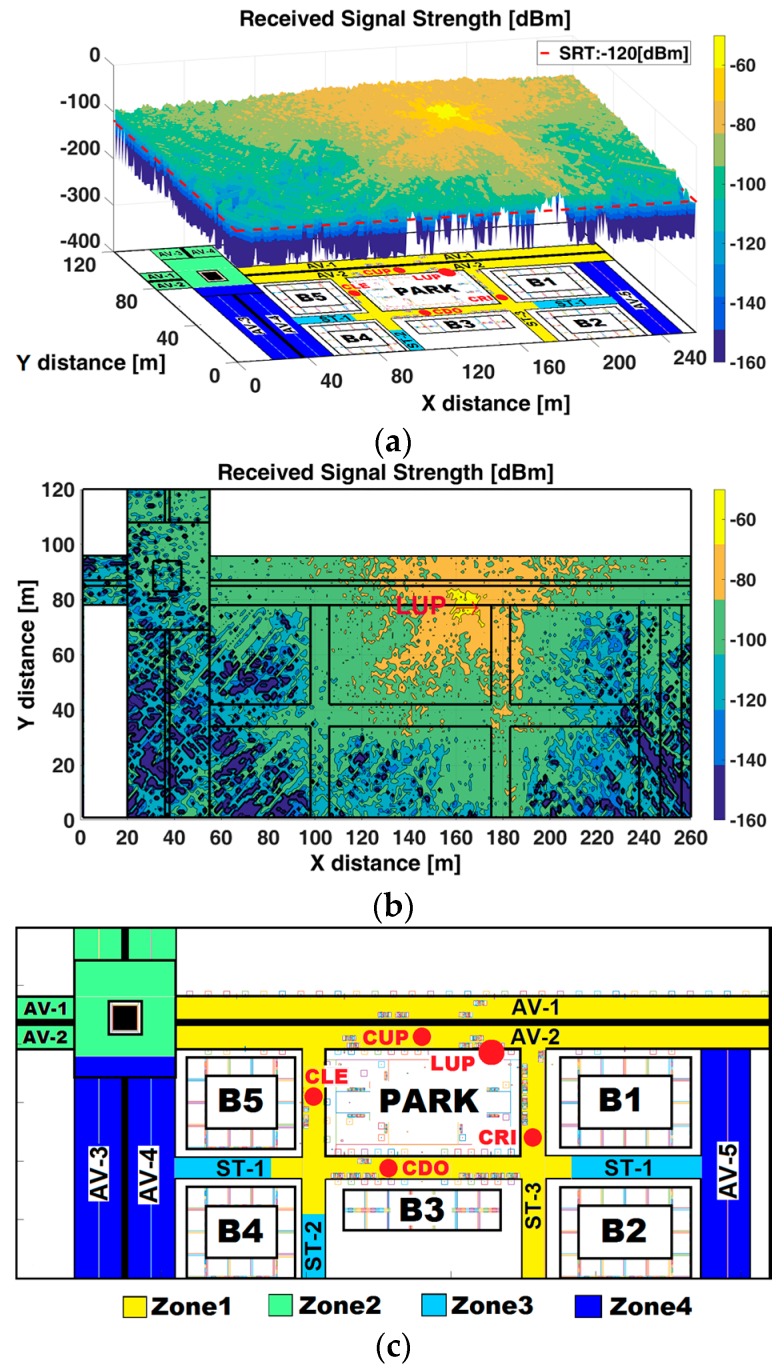
(**a**) RSS surf-plot; (**b**) RSS contour map; and (**c**) RSS zone map, at z-plane of 1.5 m.

Figure 2a illustrates a general RSS surf-plot, Figure 2b shows a RSS contour map at z-plane of 1.5 m—the height of Rx antenna emplacement in cars (see Figure 1b for car’s positions)—and Figure 2c depicts a RSS zone map according the received RSS level.

From the analysis of Figure 2, it is possible to identify four RSS zones:

1. Zone 1 (yellow colored in Figure 2c) is mainly under LoS condition without obstruction by buildings, however some obstacles (e.g., trees, cars, lamppost) can cause obstruction. These types of partially obstructed links are sometimes referred to as Quasi-Line-Of-Sight (QLoS) [43]. The average RSS is above −100 dBm, thus V2I communication is feasible. This zone encompasses avenues AV-1 and AV-2 (LoS), the street ST-1, ST-2 around the park (QLoS), and ST-3 (QLoS).
2. Zone 2 (light-green colored in Figure 2c) is under LoS, without obstruction by buildings, but with the presence of a roundabout, which causes degradation and high fluctuations in the RSS. The RSS is fluctuating between −100 dBm and −140 dBm. The conditions for V2I are degraded. This zone comprises the roundabout (LoS), AV-1, AV-2 before the roundabout and AV-3, AV-4 after the roundabout.
3. Zone 3 (light-blue colored in Figure 2c) is under NLoS conditions caused by buildings. The position of buildings can generate a corridor-configuration at some street segments, where the waveguide effect [44,45] is produced. The average RSS fluctuates between −100 dBm and −120 dBm. The conditions for V2I communications are degraded. This zone encompasses street ST-1 between B5-B4 and B1-B2 and, ST-2 between B4 and B3.
4. Zone 4 (blue colored in Figure 2c) is under NLoS caused by buildings. The average RSS is below −120 dBm with high fluctuations. The conditions for V2I communications are unfeasible. This zone encompasses avenues AV-3, AV-4 and the AV-5.

In Figure 2, one can observe the impact that distance (Rx-Tx); factors such as density and material (different electromagnetic properties), geometry and location (the buildings define the LoS and NLoS conditions); and places such as roundabout and intersections (deteriorate the RSS irrespective of distance) have on the propagation phenomenon. In previous work [22], the impact of the frequency on the propagation in this scenario was shown, the mean RSS at 5.9 GHz is below that at 2.4 GHz. The NLoS caused by buildings generate high path loss and notorious fluctuations in the RSS at AV-3, AV-4 and AV-5. The corridor configuration at streets ST-1 and ST-2 (light-blue colored) causes the waveguide effect when RSU rays impinges upon the lining walls of the buildings where the location of the RSU (angle of incidence) together with factors such as the width and length of the streets define the magnitude of the waveguide effect. A detailed RSS analysis of the propagation along streets and avenues is addressed in the next subsection.

With the RSS information from Figure 2, some alternatives of RSU deployment to ensure the V2I communication from the point of view of connectivity (RSS above RST) could be proposed. In terms of radio planning analysis and coverage/capacity estimations, one alternative could be to consider at least four Tx distributed as follows:

1. Two Tx could be in opposite corners of the park, the first at the intersection of AV-2 and ST-3, and the second at the intersection of ST-1 and ST-2. This configuration ensures RSS above RST at AV-1, AV-2, ST-1, ST-2, and ST-3.
2. One Tx could be located at strategic surrounding area of the roundabout to allow coverage for the roundabout, AV-3, AV-4 and part of AV-1 and AV-2.
3. One Tx could be placed at intersection of AV-2 and AV-5 so that ensures the coverage for AV-5 and part of AV-1.

### 4.2. Large-Scale Spatial Path Loss Characterization

The coverage of WSN in the design phase of V2I communication depends on the accurate estimation of the propagation model parameters. Any error reduction between theoretical or predicted by simulation versus measured RSS will have a significant impact in the coverage estimation, identification of undesired power losses, RSU arrangement, size and performance of the WSN network, which have direct implication in quality of service and project viability. The 3D-RL technique is a well-recognized tool for this purpose.

**Figure 3:**
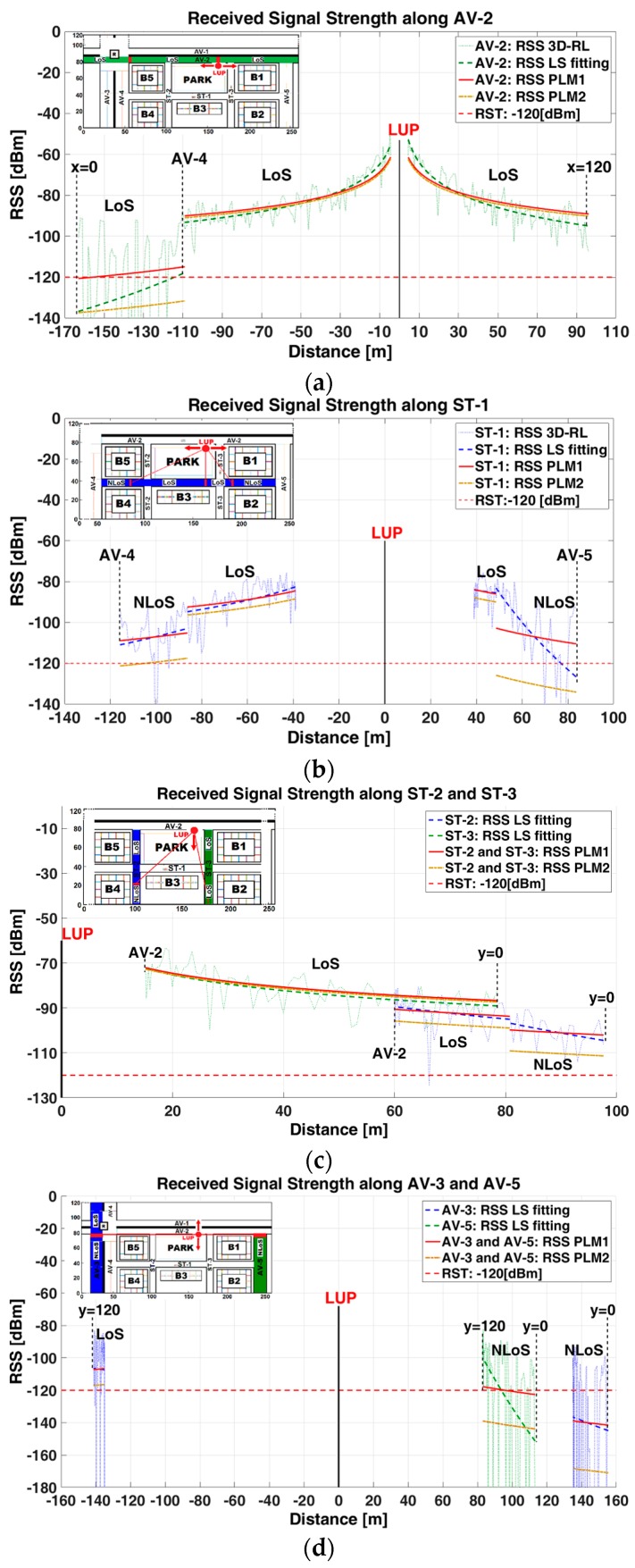
RSS along: (**a**) AV-1; (**b**) ST-1; (**c**) ST-2 and ST-3; and (**d**) at z-plane = 1.5 m.

Figure 3 depicts a detailed spatial representation of the power received at Rx height (z-plane of 1.5 m) along the avenues and streets. The LoS and NLoS conditions are identified considering the relative spatial distance Tx-Rx (left, right, up, down).

There are four different RSS representations in each image of Figure 3:

(1) RSS (dotted-line): 3D-RL technique, is the representation of the raw data obtained from the scenario simulation, organized in an array of 20 matrices (z-plane), each one with dimension of 260 × 120 (x-plane and y-plane).
(2) RSS (dashed-line): Fitting of the RSS raw data using a first order polynomial that represents the tendency-line of the raw data. This Least Squares (LS) fitting is robust to minimize the effect of some outliers in the RSS raw data and let us visualize the RSS propagation behavior. It constitutes the comparison point to measure the goodness-of-fit with any path loss model.
(3) RSS (continuous-line): Path loss model (PLM1) [46] is used for comparison purposes with the 3D-RL results. This PLM describes the random shadowing effects over a large number of measurements that have the same Tx-Rx separation, but different levels of clutter on the propagation path. The received power $P_{r}$; (RSS in this work) is defined by
  (3) $$P_{r}\left(d\right)\left[\mathrm{dBm}\right]=P_{t}\left[\mathrm{dBm}\right]+G_{t}\left[\mathrm{dB}\right]+G_{r}\left[\mathrm{dB}\right]-\left[\mathrm{PL}\left(d_{o}\right)\left[\mathrm{dB}\right]+10\mathrm{nlog}10\left(\frac{d}{d_{o}}\right)\left[\mathrm{dB}\right]+X_{\sigma }\left[\mathrm{dB}\right]\right]$$
  (4) $$\mathrm{PL}\left(d\right)\left[\mathrm{dB}\right]=-10\log_{10}\left(\frac{\lambda^{2}}{{\left(4\pi \right)}^{2}d^{2}}\right)$$
(4) RSS (dashed-point-line): Path loss model (PLM2), [12] is used for comparison purposes with the 3D-RL results. This analytical PLM2 does not consider either a free space reference distance (d_o_), or free space path loss PL(d_o_). The $P_{r}$ is defined as
  (5) $$P_{r}\left(d\right)\left[\mathrm{dBm}\right]=P_{t}\left[\mathrm{dBm}\right]+G_{t}\left[\mathrm{dB}\right]+G_{r}\left[\mathrm{dB}\right]-10\log_{10}\left(16\pi^{2}\frac{d^{n}}{\lambda^{n}}\right)$$

For PLM1 and PLM2, $P_{t}$ is the transmitted power defined at −10 dBm. $G_{t}$ and $G_{r}$ are the antenna gains equal to 3.74 dB. The free space reference distance is d_0_ = 1 m. The free space path loss $\mathrm{PL}\left(d_{o}\right)$, was calculated using Equation (4), where d = d_0_ = 1 m. X_σ_ is a zero-mean Gaussian distributed random variable required for Equation (3) with standard deviation σ (STD), and the path loss exponents (PLE = n) required in Equations (3) and (5) were calculated from the 3D-RL data using Maximum Likelihood (ML) [47], according to

(6) $$\mathrm{PLE}=\frac{-\sum_{i=1}^{n}\left(P_{i}\left[\mathrm{dBm}\right]-P_{o}\left[\mathrm{dBm}\right]\right)\log_{10}\left(\frac{d_{i}}{d_{o}}\right)}{\sum_{i=1}^{n}10{\left(\log_{10}\left(\frac{d_{i}}{d_{o}}\right)\right)}^{2}}$$

(7) $$\mathrm{STD}=\frac{1}{\sqrt{n}}{\left\{\sum_{i=1}^{n}{\left[P_{i}\left[\mathrm{dBm}\right]-P_{0}\left[\mathrm{dBm}\right]+10\log_{10}\left(\frac{d_{i}}{d_{o}}\right)\right]}^{2}\right\}}^{\frac{1}{2}}$$

Table 3 shows the PLE and STD values for each street and avenue and a measure of goodness-of-fit (GOF) between the LS fitting and the PLM1, which results in better fit than PLM2, represented by the determination coefficient R^2^.

Figure 3a–d illustrates the RSS at 1.5 m height at AV-2, ST-1, ST-2 and ST3, AV-3 and AV-5, respectively, as a function of the Euclidean distance to the Tx (LUP). The left (x-axis) and top (y-axis) distances from the Tx have been spatially differentiated from the right and bottom distances, being prefixed with a negative sign. The dotted-line represents the raw RSS data generated by 3D-RL simulation. The dashed-line represents the LS fitting-line. The continuous-red-line and dashed-dot-line represent the PLM1 and PLM2, respectively.

Figure 3a illustrates the RSS at AV-2 avenue, and shows good agreement (except for the roundabout sector) between the 3D-RL and the PLM1 (R^2^ = 0.94 and 0.91 for the left and right distances from Tx, respectively) which can be explained for the optimal LoS conditions. The presence of the roundabout, which can be considered a special type of street intersection, causes a meaningful decay in the RSS becoming evident in the increment of the PLE and STD values resulting in higher path loss and RSS fluctuation that other avenue segments. The roundabout PLE = 3.76 is the highest along the AV-1 with a dramatic increment in its STD = 39.12 dB versus STD = 5.59 dB from the same AV-1 contiguous segment. The PLMs are unable to predict the RSS at the roundabout sector which is illustrated as the lack of agreement between the LS fitting and the PLM1 (R^2^ = 0). The conditions for the V2I communications are significantly degraded or are unfeasible at this sector. According to Shivaldova et al. [48], the presence of street intersections and roundabouts are impairment factors that may deteriorate the power signal irrespective of the Tx-Rx distance.

The LoS and NLoS conditions and its effect in the RSS value along ST-1 street are illustrated in Figure 3b. The obstruction due to the trees in the LoS segments causes an increment in the PLE and STD values which are higher than the reported under LoS optimal conditions in AV-1. However, the coefficient of determination evidences a good agreement between 3D-RL and PLM (R^2^ = 0.87 and 0.72, for the left and right distances from Tx, respectively). Under LoS the RSS is above the RST with average STD = 7.22 dB, which means appropriate conditions for V2I communications. Under NLoS condition there are remarkable differences between the left and right ST-1 segments. The right segment, although closer than the left segment to the TX, shows higher PLE and STD values (PLE = 3.25 and STD = 22.43 dB in front of 2.26 and 18.37 dB of the left side respectively) which means higher path loss and signal fluctuations. The PLMs are unable to predict the behavior of the RSS at the right NLoS segment (R^2^ = 0.31). The ST-1-right NLoS segment is more obstructed by the building B1 than the left-ST-1 NLoS segment by the building B5. This behavior is highly influenced by the location of the Tx, which determines the incidence angle of the emitted rays that impinges on the buildings walls and defines the intensity of the waveguide effect in the corridor configuration at ST-1 street. The propagated rays of the transmitter are better guided in the corridor between buildings, where the incidence angle of the main ray is less obstructed. This phenomenon must be taken into account to define the RSU’s placements in the V2I environment. Under NLoS conditions, high fluctuation in RSS causes values below RST and jeopardizes the conditions for V2I communications.

Figure 3c depicts the propagation at ST-2 and ST-3 streets. The ST-3 street is under LoS with PLE = 2.07 and STD = 5.34 dB. The PLMs show good agreement with the LS fitting 3D-RL with R^2^ = 0.90. The V2I communication is workable at this segment. The RSS at ST-2 street under LoS is influenced by obstacles in the park sector (QLoS) which causes a PLE = 2.40 higher than in free-space and a STD = 7.35 dB. The GOF (R^2^ = 0.77) indicates an acceptable level of agreement with PLM1. The V2I communications are feasible considering some outliers in the 3D-RL raw data that can affect the results. Under ST-2 NLoS condition, there is a slight increment in the PLE = 2.72. The GOF (R^2^ = 0.48) for PLM1 indicates the low agreement with the LS fitting mainly due to the phenomena of diffraction in B3 and reflection on B4. The conditions for V2I communications are in the limit, near to the RST. The waveguide effect is not present in this corridor-configuration segment.

Figure 3d illustrates the propagation at AV-3 and Av-5 avenues. Buildings, as the most significant obstacles for the LoS in this urban scenario, cause the highest PLE values: 4.27 and 3.64 for AV-3 and AV-5, respectively. However, the most dramatic increment is represented by the STD: 42.76 dB and 38.01 dB, respectively. The PLMs are unable to represent the RSS behavior with R^2^ values of 0.49 and 0.18 for AV-3 and AV-5, respectively. The RSS is below −120 dBm with high fluctuations and totally degraded V2I communications conditions.

In general, these results are coherent with those obtained in Figure 2 and reported in the literature [46,49]. PLE ranges from 2.07 to 3.25 for LOS and QLOS, and 3.64 to 4.27 for NLOS; higher values have been derived from building obstruction. V2I communication is suitable at zone 1, zone 2 (excepting the roundabout area) and zone 3 (LoS and left-NLoS). Under LoS, PLM1 is more accurate than PLM2 to predict the RSS, nonetheless, under NLoS caused by buildings, both PLMs are incapable to predict the RSS behavior. The emplacements of the Tx have great influence on the creation of the LoS, NLoS conditions and the corridor-configuration with its inherent waveguide effect.

### 4.3. Multipath Metrics

The analysis of multipath metrics such as Power Delay Profile (PDP), Mean excess delay, Root Mean Square Delay Spread (RMS delay spread) and Coherence Bandwidth (CB) has great importance in WSN to design some general guidelines for deployment. To mention, the cyclic prefix (CP) of adaptive algorithms aimed to increase the spectral efficiency is determined by the maximum excess delay or by the RMS delay spread of that environment, [50]. Environments with high RMS delay spread cause inter-symbol interference (ISI), and affect the guard interval in OFDM systems; similarly, small coherence bandwidth affects the OFDM pilot spacing [51]. The multipath metrics must be understood and determined for different environments to develop a proper scheme for communications requirements.

#### 4.3.1. Power Delay Profile (PDP)

**Figure 4:**
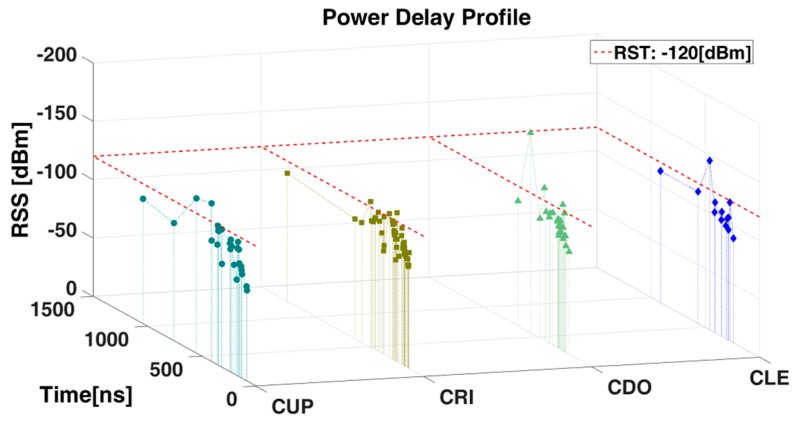
Power Delay Profile.

The PDP quantifies the number and severity of power rays (echoes) in the wireless channel and is used to calculate various multipath statistics such as mean excess delay, RMS delay spread and maximum excess delay. Figure 4 illustrates the power delay profile of Tx (LUP) when the propagated rays impact upon the Rx: CUP, CRI, CDO and CLE, located at 24.104 m, 32.802 m, 50.359 m and 52.386 m from LUP, respectively.

Figure 4 shows a large number of power rays (echoes) in a time span of 100 to 1500 ns, wich are the result of the multipath channel defined by the geometrical position between the transmitter and receiver, and the surrounding physical environments. The first ray arrives at 100 ns to CUP (located at 24.104 m from Tx) and at 200 ns to CLE (located at 52.386 m from Tx) which show the influence of the Tx-Rx distance in the arrival time of the first ray to the Rx. The time span evidence the ocurrence and duration of reflected, refracted, and difracted rays that arrive to the specific point. The density of rays is related to the Tx-Rx vicinity and the LoS conditions: higher density of rays with RSS above −120 dBm are present in CUP and CRI compared to CDO and CLE. In previous work [22], it was shown that higher frequencies (5.9 GHz vs. 868 MHz) imply higher time dispersive effects.

#### 4.3.2. Mean Excess Delay, Root Mean Square Delay Spread and Coherence Bandwidth

**Figure 5:**
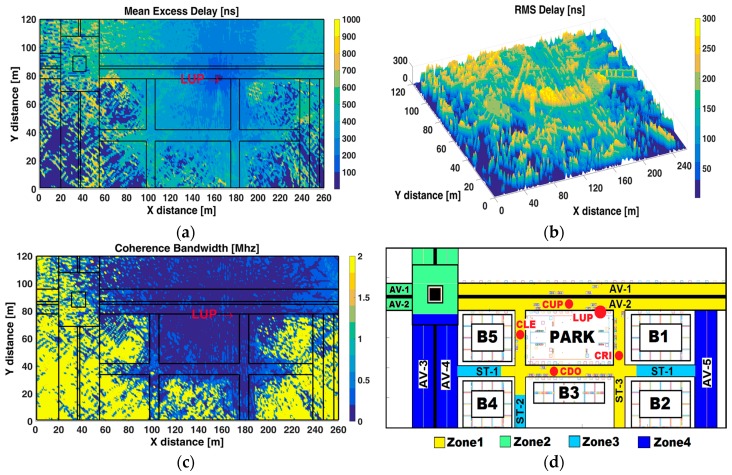
(**a**) Mean Excess Delay; (**b**) RMS delay spread; (**c**) Coherence Bandwith; and (**d**) RSS zone map.

The mean excess delay—the first moment of the PDP—and the RMS delay spread—the square root of the second central moment of PDP—together quantify the time dispersive properties of multipath channels and are measured relative to the first detectable signal arriving at the receiver. The CB is the range of frequencies over which two frequency components have a strong potential for amplitude correlation, and it is a defined relation derived from the RMS delay spread [46]. The frequency correlation function was considered above 0.9 for the analysis of CB. Figure 5a–d depicts the mean excess delay, RMS delay spread, coherence bandwidth, and RSS zone map, respectively, all of them at z-plane of 1.5 m.

According to Figure 5a, the mean excess delay is highest at zone 4 (NLoS) due to the distance and the obstruction caused by buildings, followed by zone 2 (LoS) where the roundabout causes great fluctuations in the received signal, then zone 3 under NLoS condition where the corridor-configuration causes reflections of the power signal, and finally the lowest mean excess delay is at zone 1 (LoS). It is important to note that lower values of mean excess delay into and behind buildings (B1, B2, B3, B4, and B5) denote the absence of signal due to the phenomena of absorption and reflection of propagated signals by the concrete walls and glass windows. This finding is in agreement with that reported in [52], where it is stated that the NLoS scenarios have much higher mean excess delay than LoS scenarios as the strong LoS components tend to dominate the PDP, and the transmitted signals in NLoS environments will encounter many reflections along their path to the receiver.

Figure 5b shows high RMS delay spread values common in urban scenarios due to the large number of reflections, of varying length, in this multipath environment. The vicinity to Tx, the presence of different obstacles (trees, pedestrians, lampposts, cars, benches, and grass) and the LoS conditions cause the highest RMS delay spread values at zone 1 (Park area has the higher values at zone 1), while NLoS conditions, farther distances to the Tx and the presence of factors such as the roundabout cause lower or the absence of RMS delay spread values. Zone 1 shows the highest values, followed by zone 2, then zone 3. The lowest values at zone 4 indicate the absence of propagated signal due to aforementioned factors.

Figure 5c depicts the CB values and shows its inverse relationship with the RMS delay spread. Zone 1 presents the lowest CB due to its strong multipath environment which means high channel occupancy by the Tx, followed by zone 2 (except for the roundabout). On the other hand, the highest CB value at zone 4 is an indicator of channel availability for other transmitters given that according to Figure 2b the RSS is below to the RST and the V2I communication is unfeasible, leaving the channel available for its use at that zone. CB analysis must be complementary with the RSS analysis in order to contrast the channel availability with the received power: complete NLoS environments could lead to high CB, however, the wireless communication with the Tx is not always achievable.

**Figure 6:**
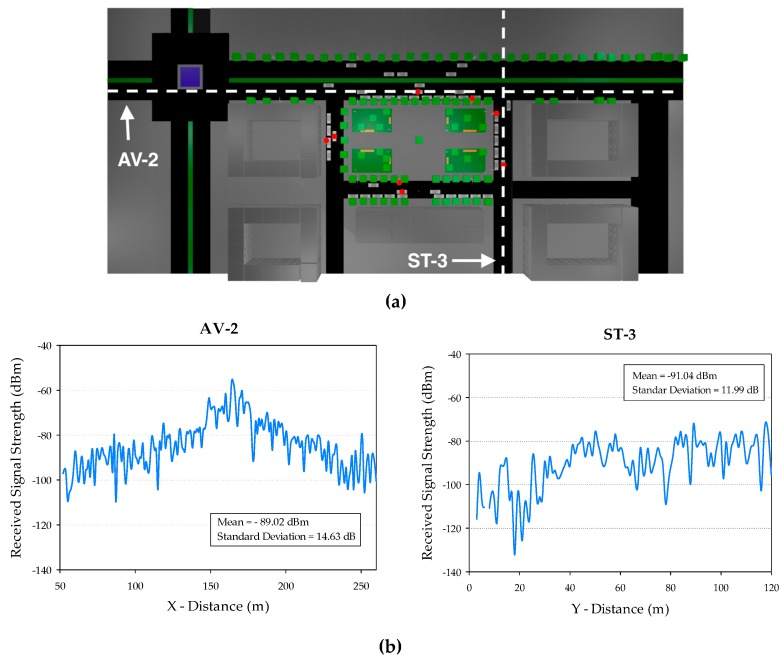
(**a**) Location of AV-2 and ST-3; (**b**) estimation of movement related standard deviation loss component for both locations.

In order to gain insight on the impact of movement in the case of vehicular transceiver, variations of received power level can be superimposed as a loss component related to received power standard deviation, as a direct function of velocity. Figure 6 presents the results obtained for the specific case of spatial variation in AV-2 and ST-3, which can be extended without loss of generality to the complete test scenario. Applicable standard deviation values, which are superimposed as an additional loss component to the values obtained by the 3D Ray Launching code are depicted in Table 4, with values in the 10.8–14.7 dB range for the complete scenario.

## 5. Measurement Validation

An RSS measurement in zone 1 was performed to establish a comparison versus the 3D-RL technique and the two analytical PLM. The transmitter antenna Tx was connected to a signal generator at 5.9 GHz and has been located at the upper lamppost LUP (x = 164 m, y = 78 m, z = 3.5 m) at 3.5 m height. The receiver antenna was placed in the compartment of a sedan-car, just below the front windshield at 1.30 height. The Tx and Rx antennas were ACA-4HSRPP-2458 from ACKme Networks (Los Gatos, CA, USA), both omnidirectional, with a gain of 3.74 dB. The employed signal generator was a portable N1996A (Agilent Technologies, Santa Clara, CA, USA) unit and the spectrum analyzer was an Agilent N9912 Field Fox. The measurements were performed with 100 MHz bandwidth at 5.9 GHz frequency and the measurement time at each point was 60 s. RSS in each point is the highest peak (Max-Hold function) of power shown by the spectrum analyzer.

**Figure 7:**
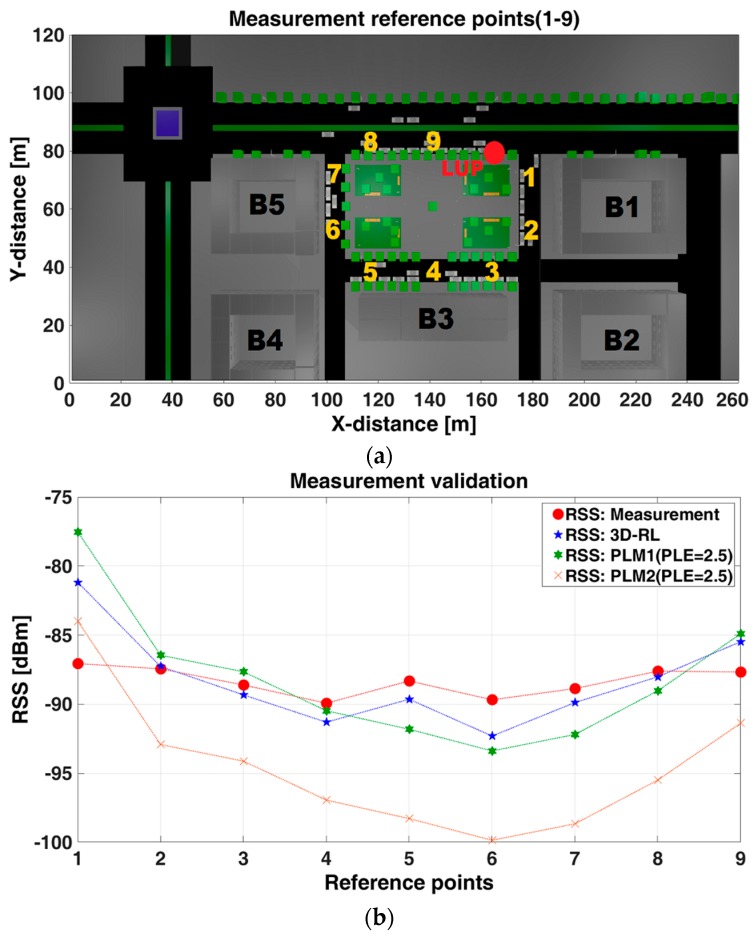
(**a**) Measurement reference points (1–9); and (**b**) measurement validation.

Figure 7a shows the nine points used as reference for the measurements campaign at zone 1: points 1 and 2 are along ST-3, points 3–5 are along ST-1, points 6 and 7 are along ST-2 and points 8 and 9 are along AV-2. Figure 7b depicts a comparison between measurements versus the 3D-RL technique and versus PLMs defined in Section 4.2.

RSS values estimated by 3D-RL were obtained taking as reference the same spatial coordinates of the points (real measurements) and evaluating the resulting distance in a first order polynomial that represent a LS fitting of a 10 m street or avenue segment that include the same coordinates in the three-dimensional mesh of cuboids in which the scenario was divided. PLM1 was evaluated according Equations (1) and (2), and PLM2 was evaluated according Equation (3).

Figure 7b shows better agreement of the 3D-RL than PLMs with the measured points, which means more accurate representation of propagation phenomena. A mean error of 1.75 dBm and standard deviation of 2.54 dB corresponds to the 3D-RL simulation, while mean error of 3.0 dBm and standard deviation of 4.18 dB correspond to the PLM1 when compared against measurements. A mean error of 6.96 dBm and standard deviation of 4.18 dB results for PLM2.

These results (zone 1) validate the 3D-RL technique as more accurate than the used PLMs in this urban environment. The effect of the obstacles in the propagation is more accurate represented with less variability in comparison with measurements. PLM1 shows better fit with the measurements than PLM2. The applicability of this results and the accuracy needs of the mean error or standard deviation will be related with the particular V2I service or application requirement.

As it can be seen from the results, received power level values are within the sensitivity range of conventional wireless transceivers, with values above −100 dBm. In terms of coverage restrictions, node density presents a variability in the order of 1–2 nodes/10^4^ m^2^, whereas in terms of capacity calculation, it is a direct function of the number of active transmissions within the scenario under analysis. Taking into consideration potential maximum bit rate allocated per user (i.e., 250 kbps), a superimposed WLAN concentrator could handle the complete traffic of the scenario. However, if traffic is handled by 802.15 gateways, traffic type comes into consideration, in terms of allowed latency and required effective bit rate. In this case, node density will increase, in the range of 1 node/10^3^ m^2^, which would be practically implemented by introducing infrastructure nodes in urban furnishings, such as traffic lights, lamp posts or traffic signals. In the case that bit rate is increased (e.g., Bluetooth BLE beacons or WLAN communication), coverage and capacity estimations would be symmetric, leading to node densities in the order previously stated.

It also worth noting that an increase in transceiver density can be directly considered, given that each connection can be independently modeled and superimposed to overall estimations, in terms of received power estimations as well as time domain components, given that in principle their operation is uncorrelated. Overall effect of transceiver node density increase is requirement of lower transmit power in order to reduce overall interference and hence, increase coverage/capacity ratios.

## 6. Application

In order to provide insight in overall system performance, a VANET testbed has been implemented and its operation analyzed. We have developed an application that takes advantage of the coexistence of WSN, V2V, V2P and V2I networks. The application includes SesToCross, an advanced driver assistance system (ADAS) described in [52], an ad-hoc WSN in charge of environmental pollution monitorization, an air quality monitoring service provided by the Government of Navarre, and also a collaborative APP for pedestrians. SesToCross is a V2V system based on a semantic expert system devoted to the intelligent management of crossroads where just vehicles are involved. We have added an IEEE-802.15.4-based WSN of 10 Waspmote v1.1 nodes placed around the campus of the Public University of Navarre (Campus de Arrosadía) which are connected to a Meshlium Sensor Gateway in charge of data collection and its transmission, through the Wi-Fi network of the campus, to the system. Data are also obtained from a web service [53] and from an APP developed for the Android platform. The APP allows collaborative notification of traffic congestion, proposes alternative routes based on user preferences, weather conditions and other customizable variables. The APP allows geo-spatial location and visualization.

Wireless Sensor network deployment has been performed following the guidelines previously presented, in order to guarantee coverage/capacity restrictions. Taking into account that preliminary tests involve low density as well as low bit rate (due to the inherent traffic information exchanged), node location is determined on compliance with receiver sensitivity levels as a function of interference conditions [54]. Selected node density has been 1 node/10^4^ m^2^, corresponding to the most restrictive value in terms of network deployment, as previously derived from coverage/capacity estimations.

**Figure 8:**
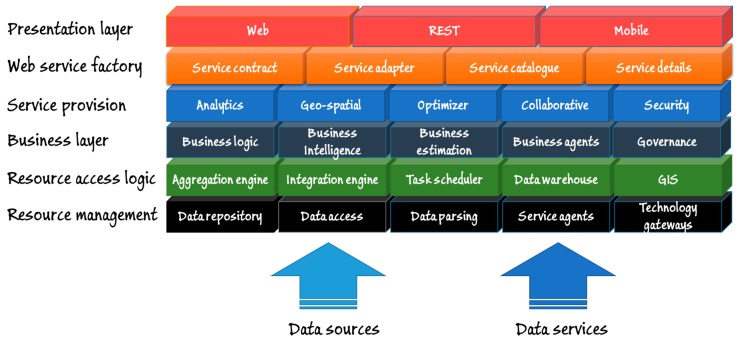
Architecture of the system.

Figure 8 depicts the architecture of the system, which is structured in six layers. Lower layers are related to data acquisition. Data sources and data services (data collected by the WSN, data obtained from the monitoring service and data collected from the collaborative APP) provide the resources needed to provide services at the higher layer. Resource management includes a MySQL [55] database as data repository. Data are gathered through the data access service; a web service that collects data provided by the WSN, the APP and the monitoring service. Since data come from different sources, and it arrives in heterogeneous formats, there is a conversion module in charge of data parsing before its storage into the repository. The resource management layer is completed with a module of service agents in charge of periodical and automated information collection from external monitoring and weather web services, and a technology gateway that allows the communication using IP and IEEE-802.15.4 connections. The resource access logic layer allows data processing. For such purpose, it includes a task scheduler, a geographic information system (GIS), a data warehouse and two engines devoted to data aggregation and data integration, respectively. This layer allows the use of business intelligence techniques and tools located at the next layer, since data are consistently and uniformly stored. The highest intelligence level of the system lies in the intermediate levels (Service provision and Business layer). The Business layer includes in our case a Pentaho-based business intelligence tool, as well as our own prototype of motion estimation engine. The rest of the layer allows the interaction of authorized users related to the governance of the system. The Service provision layer ensures security services as encryption and digital signature management, but also geo-spatial representation through the Google Maps API [56], and some analytic and optimization features now based on Octave [57]. Finally, the web service factory layer is devoted to provide services to users through the Presentation layer. Mobile devices can access the services through web services from web interfaces, REST APIs or mobile HTML5-basedAPPs. For such purpose, the Web service factory provides the catalogue of services, the service description (WSDL) and the service contract.

The system described above includes many subsystems and applications, and they must coexist and operate together. In order to grant this coexistence and to provide easy monitorization and management of the whole system, we have selected a Proxmox Virtual Environment (Proxmox VE) [58]. Proxmox VE is an open-source Debian-based server virtualization environment that allows the deployment and management of both containers (Linux Containers, LXC) and virtual machines (Kernel-based Virtual Machine (KVM)). Proxmox VE includes a Web console, command-line tools and a REST API. The choice of Proxmox is due to the high availability it provides, the live migration it allows, the flexible storage it brings, and also to the scheduled backup it allows. In our case, we have chosen containers with version 16.04 LTS of Ubuntu. Each relevant component of the system has its own container. For example, MySQL, Apache Tomcat, Octave and Pentaho servers have their own containers.

**Figure 9:**
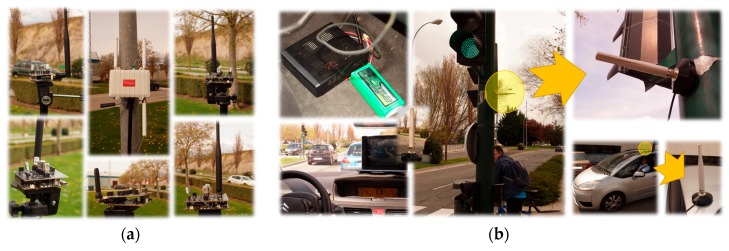
Different devices employed in order to implement the VANET WSN testbed: (**a**) infrastructure gateways and motes; and (**b**) embarked transceivers.

On the hardware side, we have used for the ADAS system of each vehicle a similar scheme to this proposed in [54], including an Intel Core i3-3450S Ivy Bridge Mini-ITX microPC, a ND-100S USB GPS dongle, IEEE 802.11 (Wi-Fi) cards Ubiquiti UBNT-SR71A operating with the Ath9k (SR71A) driver in the 5 GHz band, and a tablet. The WSN includes a Meshlium node a set of ten Waspmote v1.1 nodes, while the APP has been validated on several Android-based smartphones. Figure 9 shows the hardware used. Figure 9a depicts the WSN, where the Meshlium node (a Linux-based data aggregator) is located on the middle of the image and some Waspmote v1.1 nodes including humidity, temperature, carbon monoxide and dioxide (CO and CO_2_) and air contaminants (C_6_H_5_CH_3_, H_2_S, CH_3_CH_2_OH, NH_3_, and H_2_) sensors surround it. Figure 9b shows the ADAS subsystem, including a miniPC and its supply battery, the tablet where recommendations and advertisements are noticed, and the WiFi antennas (semaphore and vehicle).

System validation is performed using two vehicles equipped with SesToCross and the hardware described above, the WSN and the Proxmox VE infrastructure. Waspmote nodes communicate among them using IEEE 802.15.4 interfaces; the microPCs and the tablet (located inside the vehicle) communicate between them using IEEE 802.11 interfaces; the Proxmox infrastructure, the smartphones and the Meshlium node communicate using IP technologies. The routing algorithm for meshed area networks used is BATMAN Advanced [53]. Communication among vehicles and infrastructure (V2V and V2I) is performed by means of UDP datagrams. The semaphore (42.797347, −1.631630, latitude-longitude coordinates) includes a miniPC, an Ubiquiti UBNT-SR71A card and a 5 GHz Wi-Fi antenna. Wireless link distance is within the range defined by the selected node density as a function of the employed transmission rate, as previously obtained from wireless channel measurement and analysis. Sensor nodes collect the information referred to humidity, temperature and gas contamination and transfer it to the Meshlium node, which sends the information gathered by the WSN to the virtualized services using the campus Wi-Fi network of the university. The service agents of the system collect the information available from the air monitoring service [54], and vehicles interact with the rest of vehicles (directly via Wi-Fi) and with the semaphore. Both, vehicles and infrastructure may interact with the system pushing and pulling data to and from the system. Thus, information concerning contamination, weather conditions, and traffic congestion may be accessed by drivers and pedestrians through their tablets and smartphones.

**Figure 10:**
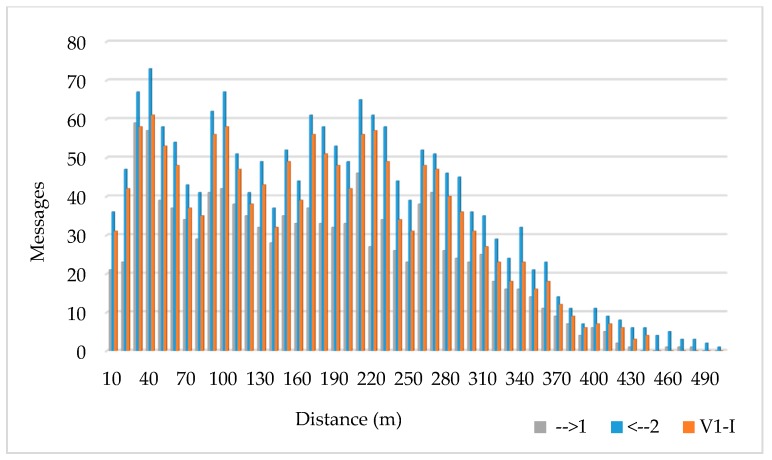
Messages transmitted by vehicle v1 and received by v2 and by the infrastructure.

Figure 10 illustrates the communication flow between the pair of vehicles and the infrastructure. The total amount of messages transmitted by the vehicle v1 (→1) is 1681, while 1163 of those messages have been received by vehicle v2 (←2), the 69.19% of the messages transmitted. If we consider the infrastructure, the node placed at the semaphore has received (V1-I) 1532 messages of the transmitted by v1, the 91.14% of the transmitted. We appreciate a low packet error rate. This is mainly because the band of 5 GHz is not occupied, in accordance with previous interference measurement and analysis and its impact in overall system performance.

## 7. Conclusions and Future Work

An in-house deterministic 3D-RL tool was used to obtain an accurate estimation of V2I radio propagation parameters such as RSS, large-scale path loss and multipath metrics in an urban scenario at frequency of 5.9 GHz, with the aim to bring insight about some radio propagation parameters essential in the design phase of WSNs deployment. The typical urban scenario was characterized in 3D high level of detail, which for the author’s knowledge, it has not been presented in previous works. It permits accurate results of radio propagation parameters that could be replicated in similar environments. Two analytical PLMs were used to establish comparison with the 3D-RL simulation, and the analysis of goodness-of-fit (R^2^) for one of them shows good agreement under LoS and certain NLoS conditions (street corridor-configuration), however they are unable to represent the NLoS propagation behind the buildings. Wireless channel estimation as well as interference analysis provide network node density, in the range of 1/node/10^4^ m^2^ and 1/node/10^3^ m^2^, as a function of coverage/capacity restrictions given by LOS/NLOS conditions within the urban vehicular scenario.

Factors such as Tx-Rx distance, placement and height of RSUs and obstacles in the LoS will have profound impact in the V2I channel propagation. Phenomena such as shadowing, reflection and refraction causes an increment in the PLE and STD values consequently causing higher path loss and large RSS fluctuations. Factors such as the roundabout cause sensitive increment in the PLE and STD values, irrespective of the distance. The NLoS conditions caused by buildings generate the highest values of PLE and STD and the V2I communication is not possible. Streets with NLoS condition and corridor-configuration (between buildings), where the waveguide effect drive the waves along the corridor, are prone to offer proper conditions for the signal propagation. At least four RSUs located at specific street intersections are needed to provide optimal V2I wireless communication in terms of coverage. The analysis of the multipath metrics shows a multipath environment evidenced in the PDP with higher mean excess delay in the NLoS than the LoS areas. High RMS delay spread values are registered in this urban scenario mainly near the Tx. On the other hand, high CB values are present in the roundabout and behind buildings which means channel availability for other transmitters. The analysis of multipath metrics must be complimentary with the analysis of the RSS in order to identify the conditions for V2I communication. Time domain parameters have also been estimated, which are directly related with fast fading components given by multipath propagation and that, provided the wireless system under consideration, determine equalization or adaptive modulation and coding schemes in transceiver operation. The analysis with the aid of hybrid deterministic code based on 3D Ray Launching enables evaluation of complex vehicular scenarios with affordable computational cost, opening a path for the analysis of issues in the near future such as scalability or heterogeneous network operation.

Measurement validation conditions evidence the better accuracy of the 3D-RL technique compared to the analytical PLMs. A mean error of 1.75 dBm and standard deviation of 2.54 dB corresponds to the 3D-RL simulation when compared with measurements. The effect of the obstacles in the propagation is accurately represented. Under NLoS conditions is expected higher error for the PLM that 3D-RL. Full system validation has been provided by implementing a real VANET WSN testbed, in which the complete software architecture and the set of V2I devices have been implemented and deployed.

As a future work, an extensive measurement campaign should be considered for the urban scenario in order to contrast the 3D-RL simulation, PLM and test-field measurements under NLoS conditions. The identification and characterization of significant areas (i.e., different density of obstacles such as buildings, vegetation, etc.) could lead us to the proposal of an empirical or statistical propagation model that accounts for more environmental variables with the aim of precise results. The zones with high CB and low RSS levels where the V2I communication is unfeasible could be of special interest in research fields such as cognitive radio and V2P, among others. The detailed and quantitative impact of street intersections and roundabout on the RSS have special interest for V2V communications. Massive V2I WSN deployments are not only technological matters, but also ones for governments, manufacturers and customers. The offer of developing services and applications for which customers are willing to pay, car manufacturers are willing to offer and government policies are willing to permit will define the future deployment of V2I technology.

## Figures and Tables

**Table 1:** Points of interest.

Description	Abbreviation	Position (x, y, z) [m]
Main Avenues	AV-1/AV-2/AV-3/AV-4/AV-5	(x, 93, 0)/(x, 82, 0)/(29, y ,0)/(47, y, 0)/(247, y, 0)
Streets	ST-1/ST-2/ST-3	(x, 39, 0)/(54, y, 0)/(130, y, 0)
Transmitter antenna	Tx (LUP)	(164, 78, 3.5)
Car antennas	CUP/CRI/CDO/CLE	(91, 82, 1.5)/(130, 50, 1.5)/(84, 38, 1.5)/(54, 62, 1.5)
Buildings/Park	B1, B2, B3, B4, B5/Park	Not applicable.

**Table 2:** Simulation parameters.

Parameters	Values
Transmitter (Tx): Tx. Power/Gain/Frequency/Height	−10 dBm/5 dB/5.9 Ghz/3.5 m
Receiver (Rx): Rx. RST/Gain/Frequency/Height	−120 dBm/5 dB/5.9 Ghz/1.5 m
Antenna Polarization	Omnidirectional
3D Ray tracing resolution	1 degree
Scenario size/Unitary volume analysis	260 m × 120 m × 20 m/Cuboids of 1 m

**Table 3:** Path loss exponent (PLE) and standard deviation (STD).

Description	PLE (n)	STD (σ) [dB]	LS vs. PLM1 GOF (R^2^)
AV-2 (along x-axis)			
Roundabout (LoS)/left-Tx * (LoS)/Tx-right (LoS)	3.76/2.13/2.22	39.12/5.59/6.59	0/0.94/0.91
ST-1 (along x-axis)			
Between B4-B5 (NLoS)/Left-Tx (LoS)/	2.96/2.30/	18.37/7.22/	0.72/0.87/
Tx-Right(LoS)/Between B1-B2 (NLoS)	2.26/3.25	7.21/22.43	0.72/0.31
ST-2 (along y-axis)			
Park(LoS)/Between B3-B4 (NLoS)	2.40/2.72	7.35/6.28	0.77/0.48
ST-3 (along y-axis) (LoS)	2.07	5.432	0.90
AV-3 (along y-axis)			
Roundabout(LoS)/Behind B4-B5 (NLoS)	2.76/4.27	31.63/42.76	0/0.49
AV-5 (along y-axis) (NLoS)	3.64	38.01	0.18

* Transmitter located at LUP (x = 164, y = 78, z = 3.5) [m].

**Table 4:** Movement Related Standard Deviation Estimations.

Avenue/Street	Mean (dBm)	Standard Deviation (dB)
AV-1	−88.70	10.881
AV-2	−89.07	14.63
AV-3	−106.25	13.03
AV-4	−104.26	11.73
AV-5	−97.37	11.71
ST-1	−97.16	11.58
ST-2	−106.16	13.67
ST-3	−91.04	11.99
